# The efficacy and safety of PD-1/PD-L1 inhibitors versus chemotherapy in patients with previously treated advanced non-small-cell lung cancer: A meta-analysis

**DOI:** 10.1097/MD.0000000000025145

**Published:** 2021-03-26

**Authors:** Lin-guang-jin Wu, Dan-ni Zhou, Ting Wang, Jun-zhi Ma, Hua Sui, Wan-li Deng

**Affiliations:** a Department of Oncology, Putuo Hospital, Shanghai University of Traditional Chinese Medicine, Shanghai 200333; b College of Acupuncture and Orthopedics, Hubei University of Chinese Medicine, Hubei 430065; c College of Physics and Information Engineering Jianghan University, Hubei 430056; d Department of Oncology, Shuguang Hospital, Shanghai University of Traditional Chinese Medicine, Shanghai 201203, China.

**Keywords:** advanced NSCLC, chemotherapy, meta-analysis, PD-1/PD-L1 inhibitors, squamous, tumor proportion score

## Abstract

**Background:**

Immune checkpoint inhibitor therapy for non-small cell lung cancer is widely used in clinical practice. However, there has not been a systematic statistical proof of the efficacy of PD-1 inhibitors in patients with advanced cancer. This meta-analysis aims to evaluate its efficacy and related influencing factors, so as to provide a basis for clinical diagnosis and treatment.

**Objective:**

To assess the effectiveness and safety of programmed death-1 (PD-1)/PD ligand 1 (PD-L1) inhibitors versus chemotherapy as second-line or late-line treatment for patients with advanced non-small-cell lung cancer (NSCLC) via a systematic review of published randomized controlled trials (RCTs).

**Methods:**

Studies were identified through PubMed, EMBASE, and Cochrane Library electronic databases. RevMan 5.3.5 was used to analyze the data extracted from all eligible studies.

**Results:**

All 4122 eligible patients from 8 RCTs were included in this study. The meta-analysis showed that PD-1/PD-L1 inhibitors could significantly improve overall survival (hazards ratio [HR] 0.71, 95% confidence interval [CI] 0.66–0.77, *P* < .001), progression-free survival (HR 0.88, 95%CI 0.81–0.94, *P* = .01), and objective response rate (HR 2.03, 95%CI 1.66–2.49, *P* < .001) compared with chemotherapy drugs. The incidence of side effects of any grade (HR 0.34, 95%CI 0.29–0.39, *P* < .001) or grades 3 to 5 (HR 0.15, 95%CI 0.10–0.23, *P* < .001) consistently showed that PD-1/PD-L1 inhibitors were safer than chemotherapy. Furthermore, subgroup analysis based on tumor proportion score or pathology classification revealed that PD-1/PD-L1 inhibitors significantly improved overall survival compared with chemotherapy.

**Conclusion:**

As a second-line or late-line treatment, PD-1/PD-L1 inhibitors are safer and more effective than chemotherapy in patients with advanced NSCLC.

## Abstract 2

Supplemental Digital Content is available in the text

## 1 Introduction

Lung cancer is one of the most common malignancies globally.^[1]^ Non-small-cell lung cancer (NSCLC) accounts for more than 80% of cancer cases, and the 5-year survival rate of patients with this cancer is approximately 16%.^[2]^ Most (65%) patients with NSCLC are diagnosed when the cancer is locally advanced (IIIB) or metastatic (IV), and the 5-year survival rates for patients with these NSCLC types are 5% and 1%, respectively. Patients receiving palliative care suffer from a high symptom burden and from side effects of toxic treatments.^[3]^

Immune checkpoint inhibitors are a new standard of treatment for patients with advanced NSCLC without abnormalities in epidermal growth factor receptor tyrosine kinase or anaplastic lymphoma kinase genes. In the case of NSCLC, programmed death 1 (PD-1) on the surface of malignant cells binds to PD ligand 1 (PD-L1), which is expressed on activated T cells and pro-B cells, to avoid killing by immune cells and thus preventing immune surveillance. Although the introduction of PD-1 or PD-L1 inhibitors into clinical practice has revolutionized cancer treatment, consistent response and beneficial long-term results have only been observed in few patients.^[4]^ Furthermore, several treatment-related side effects have been noted following first-line therapy with PD-L1 inhibitors in combination with chemotherapy^[5]^ or PD-L1 inhibitors alone.^[6]^

With the current increase of clinical trials in this area, most of them have not been included in a systematic evaluation to accurately compare the safety and efficacy of PD-L1 inhibitors as a second- or later-line therapy with those of chemotherapy for advanced NSCLC. Therefore, the aim of this research was to investigate and analyze the latest randomized control trial (RCT) evidence regarding the efficacy and safety of PD-L1 inhibitors alone with those of chemotherapy for treating advanced NSCLC. We also investigated whether tumor pathology or PD-L1 expression determined using tumor proportion score (TPS) can affect the treatment selection for patients.

## 2 Methods

This study was based on the recommendations of the Cochrane Handbook for systematic reviews of interventions, and was conducted in accordance with the Preferred Reporting Items for Systematic Reviews and Meta-Analyses statement guidelines.^[7]^

The registered study protocol is available on PROSPERO database (identification number: CRD42020158037).

### 2.1 Database and search strategy

This meta-analysis was performed by searching the PubMed, EMBASE, and Cochrane Library databases from inception to March 2020 with no language restrictions (for details on the search strategy in EMBASE, refer to Supplemental S1, http://links.lww.com/MD/F948). The primary keywords used were as follows: “Pulmonary Neoplasms,” “Carcinoma, Non-Small-Cell Lung,” “Antineoplastic Agents,” “Antibodies, Monoclonal, Humanized,” “Advanced,” “Metastasis,” “pembrolizumab,” “nivolumab,” “durvalumab,” “atezolizumab,” “avelumab,” “Docetaxel Trihydrate,” and “randomized controlled trial.”

After the comprehensive search, the shortlisted studies were screened. Three authors independently reviewed and cross-checked the quality of the included articles and evaluated them.

### 2.2 Selection criteria

The pre-specified criteria for RCT inclusion in the study were as follows:

1) Population: Patients with advanced NSCLC that was diagnosed via pathology or other imaging modalities, with no limitations of age, nationality, sex, or race.
2) Intervention: Experimental group treated with PD-1 or PD-L1 inhibitors alone (e.g., durvalumab, nivolumab, atezolizumab, pembrolizumab, or avelumab) irrespective of dosage and duration. The control groups were administered chemotherapy alone regardless of dosage and duration.
3) Outcome: The primary outcome was overall survival (OS) and secondary outcomes were progression-free survival (PFS) and the objective response rate (ORR).

Studies were excluded if complete test conditions were not available; if they were animal experimental research, reviews, and basic research or retrospective studies, other non-randomized controlled experiments, phase I and most low-quality II studies; if the experimental group was treated with PD-L1 inhibitor combined with other drugs; if the control group was not treated with chemotherapy alone; if consistent baseline levels of patients were missing; if intervention dosage was not completely clear, and if they were republished articles.

### 2.3 Risk of bias assessment

**Figure 1:**
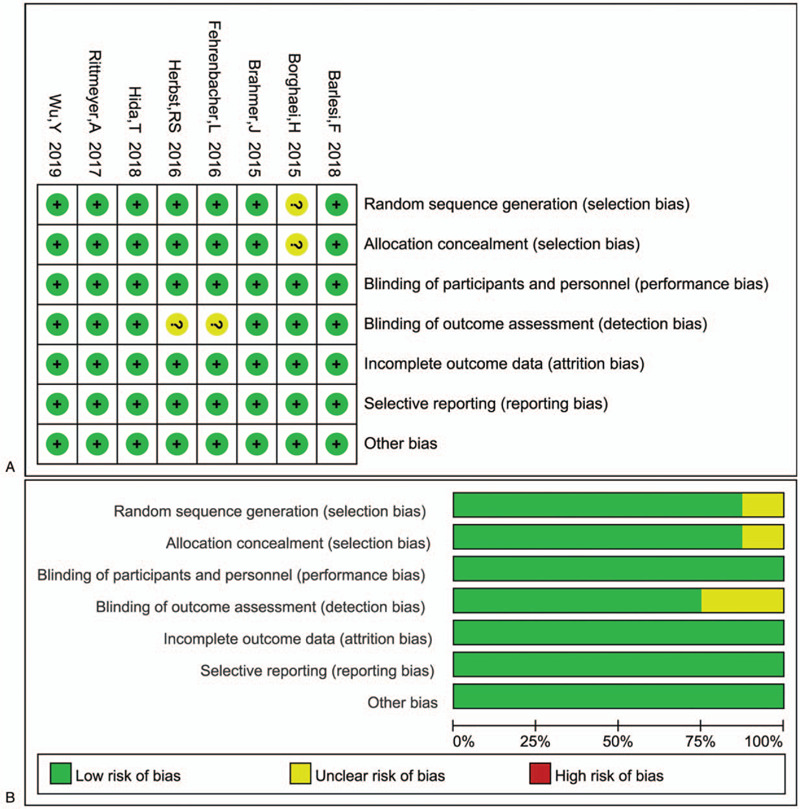
The risk of bias of included studies. A: Risk of bias summary. B: Risk of bias graph.

The Cochrane Risk of Bias Tool was used to evaluate the risk of bias.^[8]^ All included trials were assessed to have either high (green), unclear (yellow), or low (red) risk of bias based on the following 7 criteria:

1) random sequence generation,
2) allocation concealment,
3) blinding of participants and personnel,
4) blinding of outcome assessment,
5) incomplete outcome data,
6) selective reporting, and
7) other bias (Fig. 1).

Data extraction and quality assessment were performed independently by 3 authors. Any disagreements were resolved through discussion and consensus.

### 2.4 Data extraction

Three authors (LGJ Wu, DN Zhou, and JZ Ma) extracted data from 8 studies using a standardized data collection form. The reported hazard ratios (HRs) for OS and PFS and the related number of patients for the ORR were included. The following clinicopathological characteristics (Table 1) were recorded for each study: study name, trial phase, line of treatment, experimental drugs, assay developers for PD-L1 detection, observation, randomization stratified by pathology, and randomization stratified by PD-L1 expression. In addition, the inclusion and exclusion criteria, treatment received before the trial, protocol of the trial, and number of patients in each study were also recorded (Table 2).

**Table 1:** Characteristics of the included randomized controlled trial.

Study	Year	Trial phase	Line of treatment	Clinical stage	Experimental drugs	Assay developer	Observation	Randomization stratified by pathology	Randomization stratified by PD-L1 expression
Rittmeyer et al^[20]^	2017	III	2nd or later	IIIB or IV	Atezolizumab vs docetaxel	DN, Zhou	OS PFS ORR	YES	YES
Brahmer et al^[16]^	2015	III	2nd or later	IIIB or IV	Nivolumab vs docetaxel	JZ, Ma	OS PFS ORR	NO	YES
Borghaei et al^[15]^	2015	III	2nd or later	IIIB or IV	Nivolumab vs docetaxel	LGJ, Wu	OS PFS ORR	NO	NO
Barlesi et al^[14]^	2018	III	2nd or later	IIIB or IV	Avelumab vs docetaxel	JZ, Ma	OS PFS ORR	YES	YES
Herbst et al^[18]^	2016	II/III	2nd or later	IIIB or IV	Pembrolizumab vs docetaxel	LGJ, Wu	OS PFS ORR	YES	YES
Hida et al^[19]^	2018	III	2nd or later	NR	Atezolizumab vs docetaxel	LGJ, Wu	OS PFS ORR	NO	YES
Wu et al^[21]^	2019	III	2nd or later	IIIB or IV	Nivolumab vs docetaxel	DN, Zhou	OS PFS ORR	YES	YES
Fehrenbacher et al^[17]^	2016	II	2nd or later	NR	Atezolizumab vs docetaxel	DN, Zhou	OS PFS ORR	YES	YES

NR = not reported, ORR = objective response rate, OS = overall survival, PFS = progression-free survival.

**Table 2:** Clinicopathological characteristics of the eligible studies.

Study	Year	Inclusion criteria for patient selection	Exclusion criteria for patient selection	Previous treatment	Treatment	No. of patients^∗^
Rittmeyer et al^[20]^	2017	Stage IIIB or IV squamous cell or non-squamous cell NSCLC; measurable disease per Response Evaluation Criteria in Solid Tumors; ECOG PS of 0 or 1. Aged ≥ 18.	Autoimmune disease; prior therapy with checkpoint-targeted agents; prior docetaxel therapy.	One to two previous cytotoxic chemotherapy regimens	Atezolizumab (1200 mg q3w) or docetaxel (75 mg/m^2^ q3w)	850 (425/425)
Brahmer et al^[16]^	2015	Stage IIIB or IV squamous cell NSCLC; with treated stable brain metastases; ECOG PS of 0 or 1. Aged ≥ 18.	Autoimmune disease; symptomatic interstitial lung disease; systemic immunosuppression; prior therapy with T-cell costimulation or checkpoint-targeted agents; prior docetaxel therapy.	One prior platinum containing regimen	Nivolumab (3 mg/kg q2w) or docetaxel (75 mg/m^2^ q3w)	272 (135/137)
Borghaei et al^[15]^	2015	Stage IIIB or IV recurrent non-squamous NSCLC; after radiation therapy or surgical resection and had also had disease recurrence or progression; adequate hematologic, hepatic, and renal function; ECOG PS of 0 or 1. Aged ≥ 18.	Autoimmune disease; symptomatic interstitial lung disease; systemic immunosuppression; prior treatment with immune-stimulatory antitumor agents; prior docetaxel therapy.	One prior platinum-based doublet chemotherapy regimen	Nivolumab (3 mg/kg q2w) or docetaxel (75 mg/m^2^ q3w)	582 (292/290)
Barlesi et al^[14]^	2018	Stage IIIB or IV or recurrent NSCLC; disease progression after treatment with a platinum-containing doublet; adequate hematological, renal, and hepatic function; ECOG PS of 0 or 1. Aged ≥ 18.	Brain metastases; non-squamous cell NSCLC harbouring an EGFR or ALK mutation; persisting toxicity after previous treatment, or other clinically significant diseases.	One prior platinum-based doublet chemotherapy regimen	Avelumab (10 mg/kg q2w) or docetaxel (75 mg/m^2^ q3w)	529 (264/265)
Herbst et al^[18]^	2016	Stage IIIB or IV NSCLC with progression as per RECIST v1.1 after 2 or more cycles of platinum-doublet chemotherapy; PD-L1 TPS ≥ 1%; aged ≥ 18; ECOG PS of 0 or 1.	Autoimmune disease; brain metastases; carcinomatous meningitis; interstitial lung disease or history of pneumonitis; prior treatment with PD-1 checkpoint inhibitors or docetaxel.	Two or more prior cycles of platinum-doublet chemotherapy	Pembrolizumab (2 mg/kg q3w) or pembrolizumab (10 mg/kg q3w) or docetaxel (75 mg/m^2^ q3w)	1034 (345/346/343)Δ
Hida et al^[19]^	2018	Squamous or non-squamous cell locally advanced or metastatic NSCLC; disease progression during or after a platinum-based regimen; measurable disease per RECIST v1.1; tumor sample available for evaluation of PD-L1 expression; had received ≤ 2 prior chemotherapy regimens; aged ≥ 18; ECOG PS of 0 or 1.	Autoimmune disease; had received prior therapy with docetaxel, CD137 agonists, antiecytotoxic T-lymphocyte-associated antigen 4, or anti-PD L1/PD-1 therapies.	One or two prior platinum-based chemotherapy	Atezolizumab (1200 mg) or docetaxel (75 mg/m^2^ q3w)	64 (36/28)
Wu et al^[21]^	2019	Stage IIIB or IV or recurrent squamous or non-squamous cell NSCLC progressing during or after 1 previous platinum-based doublet chemotherapy regimen; measurable disease per RECIST v1.1; aged ≥ 18; ECOG PS of 0 or 1.	Active autoimmune disease, symptomatic interstitial lung disease, systemic immunosuppression; with EGFR-mutation-positive tumors or known ALK receptor tyrosine kinase (ALK) translocation-positive tumors; prior treatment with an EGFR, anaplastic lymphoma kinase inhibitor, anti-tumor vaccine, immunostimulatory antitumor agent, immune checkpoint inhibitor, or docetaxel.	One or more prior platinum containing regimen	Nivolumab (3 mg/kg q2w) or docetaxel (75 mg/m^2^ q3w)	504 (338/166)
Fehrenbacher et al^[17]^	2016	Advanced or metastatic NSCLC; measurable disease per RECIST v1.1; adequate hematological; end-organ function; provided tumor specimens for central PD-L1 testing on formalin-fixed paraffin-embedded sections before enrolment; aged ≥ 18; ECOG PS of 0 or 1.	Active or untreated CNS metastases; history of pneumonitis, autoimmune or chronic viral diseases; previous treatment with docetaxel, CD137 agonists, anti-CTLA4, anti-PD L1, or anti-PD-1 therapeutic antibodies, or PD-1/PD-L1 pathway-targeting agents.	One or more prior platinum containing regimen	Atezolizumab (1200 mg) or docetaxel (75 mg/m^2^ q3w)	287 (144/143)

∗ Expressed as total number of patients (number of patients in intervention arm/number of patients in control arm). ΔThe trial is divided into 3 groups (number of patients in intervention arm received pembrolizumab 2 mg/kg/number of patients in intervention arm received pembrolizumab 10 mg/kg/number of patients in control arm). CNS = central nervous system, ECOG = Eastern Cooperative Oncology Group, NSCLC = non-small-cell lung cancer, PS = performance status, RECIST = response evaluation criteria in solid tumors.

### 2.5 Statistical analysis

The primary endpoint was OS and was defined as the time between diagnosis and death from any cause. It is the most recognized parameter for assessing the outcome of cancer treatment. Similarly, the United States and European oncology groups agree that OS should be the primary outcome measure in clinical research. The PFS and ORR were the secondary outcomes.^[9–11]^

Review Manager (RevMan; Version 5.3.5; The Nordic Cochrane Centre, The Cochrane Collaboration, Copenhagen, 2014), the Cochrane systematic review software, was used to systematically analyze outcomes of the 8 included studies and was used to conduct a subgroup analysis based on PD-1/PD-L1 expression (determined using TPS) and tumor pathology. The traditional method of processing time to event HRs uses the O-E/V data type to evaluate the HRs.^[12]^ The same generic inverse variance data could then be used by software conversion (for details on the hazard ratio of extraction and data conversion, refer to Supplemental S2, http://links.lww.com/MD/F949). Thus, we evaluated the hazards ratio (HR) and 95% confidence interval (CI) of OS, PFS, and ORR in each study.

We assessed the statistical heterogeneity between different trials and subgroups using the Cochrane's *Q* statistic. *I*^2^ was calculated to assess the extent of inconsistency contributing to the heterogeneity across the different studies.^[13]^ The assumption of homogeneity was considered valid for *I*^2^ ≤ 25% and *P* > .10. In this study, a fixed-effects model was used when there was no obvious heterogeneity; otherwise, a random-effects model was used.

The Begg funnel plot was used to analyze publication bias, which was considered low when the completeness and symmetry of the Begg funnel plot were high.

## 3 Results

### 3.1 Literature search

**Figure 2:**
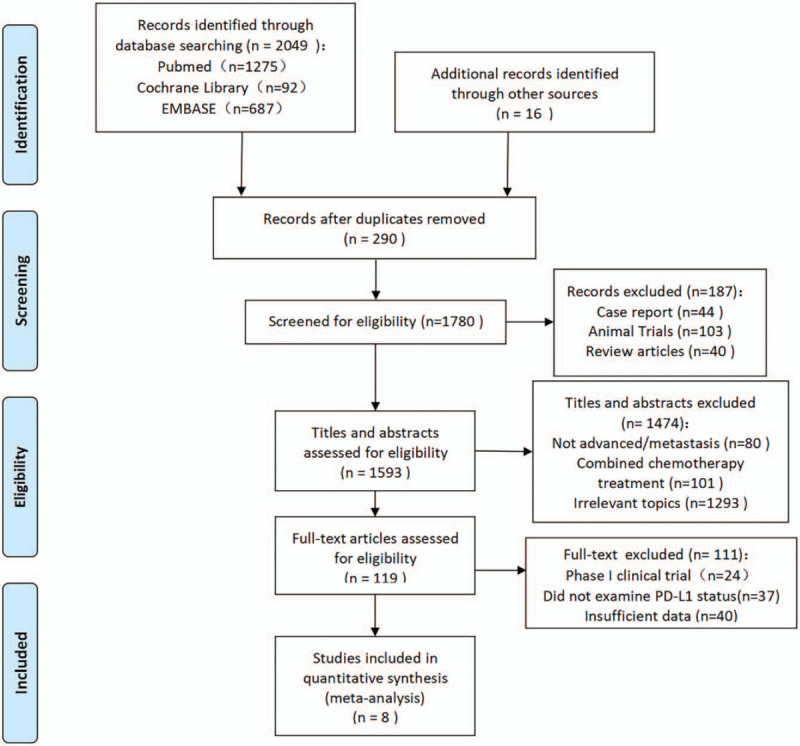
Flowchart diagram of selected randomized controlled trials included in this meta-analysis.

A total of 2049 articles were identified by the original search strategy, among which 269 articles were removed because of duplications and 187 were removed by adding qualifiers. After screening the titles and abstracts, 1474 papers were excluded; we then carefully reviewed the remaining 119 papers to shortlist 8 RCTs^[14–21]^ (Fig. 2) that met the inclusion criteria.

### 3.2 Study characteristics

A total of 4122 subjects were enrolled in the 8 studies, including 1979 in the experimental group and 2143 in the control group. The main features of the 8 studies are described in Table 1; these studies were either phase III or high quality multi-centered phase II clinical trials. All intervention groups received PD-L1 inhibitor treatment, with those in 3, 3, 1, and 1 studies receiving atezolizumab,^[14,19,21]^ nivolumab,^[15,16,20]^ avelumab,^[17]^ and pembrolizumab^[18]^ respectively. In all studies, PD-L1 inhibitors were used as second-line or later-line treatment. Apart from the studies by Hida^[19]^ and Wu,^[21]^ the remaining 6 studies described the TNM classification of tumors.

The primary endpoint in all eligible trials was OS, and the secondary endpoints were PFS and ORR. For subgroup analysis, 5 RCTs^[14,17,18,20,21]^ stratified subjects by tumor pathology and 7^[14,15,17–21]^ stratified them by PD-L1 TPS.

Random sequence generation was illustrated in all trials. Each experiment was of high quality, according to the other scoring criteria described in Figure 1.

### 3.3 Efficacy of PD-1/PD-L1 inhibitors

**Figure 3:**
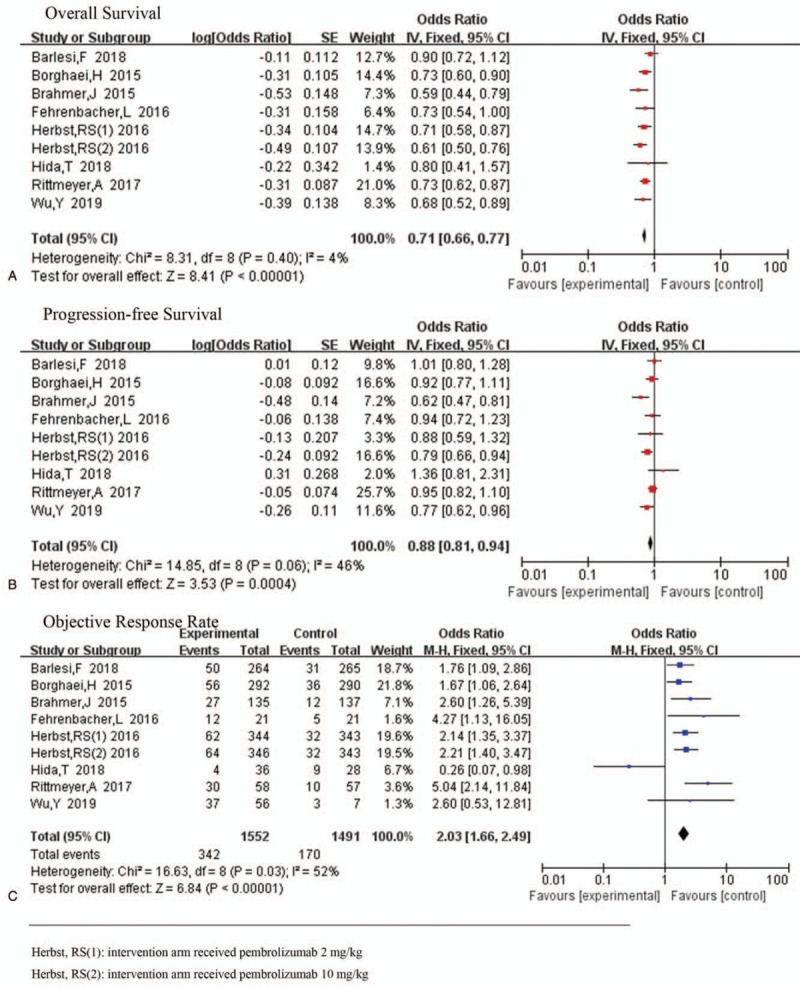
Hazard ratio of overall survival (OS), progression-free survival (PFS), and objective response rate (ORR) for patients in intervention group compared with that in the control group. The effects of therapy were calculated using a fixed-effects model in A and using a random-effects model in B and C.

In terms of OS, patients with advanced NSCLC who received PD-1 or PD-L1 inhibitors as second-line or later-line treatment had a significantly reduced risk of mortality than those who received conventional chemotherapy (HR 0.71, 95%CI 0.66–0.77, *P* < .001). No substantial heterogeneity was observed among the trials (*P* = .4, *I*^2^ = 4%) (Fig. 3A).

**Figure 4:**
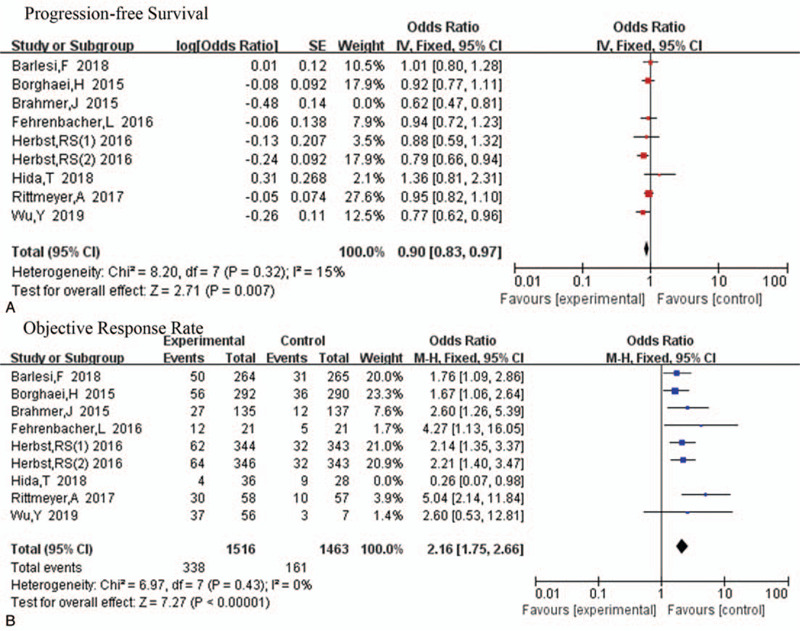
Hazard ratio of progression-free survival (PFS) and objective response rate (ORR) for patients in the intervention group compared with that in the control group after eliminating heterogeneity. The effects of the therapy were calculated using a fixed-effects model.

As shown in Figure 3B, PD-1 or PD-L1 inhibitors had the same effect on PFS as on OS (HR 0.87, 95%CI 0.78–0.97, *P* = .01). However, some statistical heterogeneity was observed (*P* = .06, *I*^2^ = 46%). By exploring sources of heterogeneity, we noted that the statistical heterogeneity disappeared after the removal of the study by Brahmer^[16]^ (*P* = .32, *I*^2^ = 15%) (Fig. 4A).

Similarly, statistical analysis also showed significant improvement in ORR in the treatment group than in the control group (HR 2.08, 95%CI 1.49–2.88, *P* < .001) (Fig. 3C). However, heterogeneity test results showed no obvious homogeneity (*P* = .03, *I*^2^ = 52%). By removing the study by Hida,^[19]^ ORR heterogeneity disappeared (*P* = .43, *I*^2^ = 0%) (Fig. 4B). Thus, it is possible that its heterogeneity was due to the small sample size, resulting in a large effect from random errors.

### 3.4 Subgroup analysis

To explore the causes of heterogeneity, heterogeneity tests were conducted based on patients TPS and pathological classification. These 2 variables were chosen as they showed the most evident differences in the included studies based on statistical analyses.

### 3.5 PD-L1 expression status

**Figure 5:**
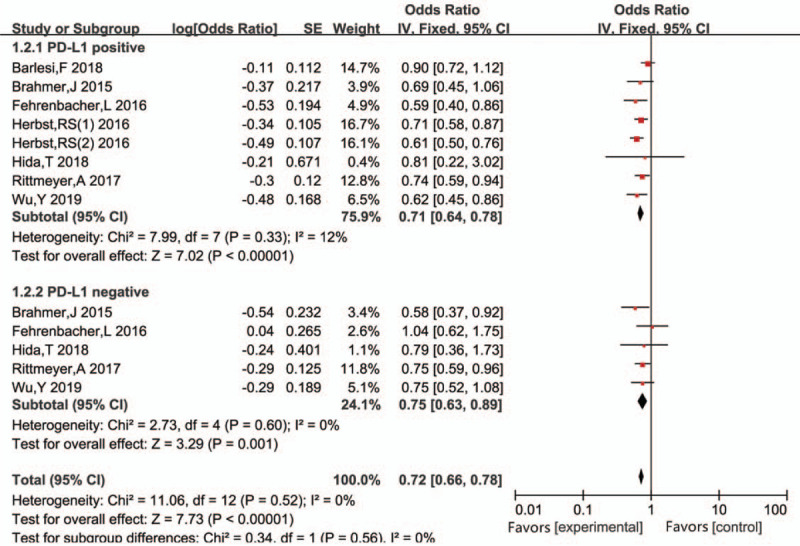
Subgroup analysis for overall survival (OS) in PD-L1-positive/-negative patients determined using TPS in the intervention and control groups.

In subgroup analyses of TPS (Fig. 5), patients with PD-L1 expression (TPS ≥ 1%) had significantly improved OS following PD-L1 inhibitor treatment than following control treatment (HR 0.71, 95%CI 0.64–0.78, *P* < .001). Notably, for patients without PD-L1 expression, PD-L1 inhibitors could still effectively reduce the risk of mortality (HR 0.75, 95%CI 0.63–0.89, *P* = .001).

No substantial heterogeneity was observed between patients with PD-L1 expression (*P* = .33, *I*^2^ = 12%) and those without PD-L1 expression (*P* = .60, *I*^2^ = 0%) based on TPS analysis. Overall, this analysis showed a consistent baseline level of PD-L1 expression, as determined by TPS, for patients included in the literature and revealed that this variable was not associated with heterogeneity in the meta-analysis.

### 3.6 Tumor pathology

**Figure 6:**
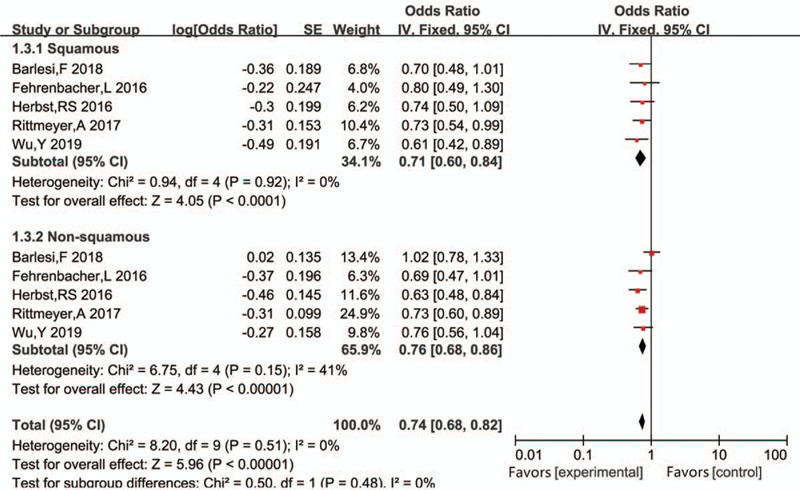
Subgroup analysis for overall survival (OS) in patients with squamous or non-squamous carcinoma between the intervention and control groups.

Subgroup analysis of tumor pathology revealed that compared with chemotherapy, PD-L1 inhibitors were effective in improving the OS of both advanced NSCLC patients with squamous cell carcinoma (HR 0.71, 95%CI 0.60–0.84, *P* < .001) and of those without squamous cell carcinoma (HR 0.76, 95%CI 0.68–0.86, *P* < .001) (Fig. 6).

Notably, statistical heterogeneity was found among the patients with non-squamous cell carcinoma (*P* = .15, *I*^2^ = 41%) but not among those with squamous cell carcinoma (*P* = .92, *I*^2^ = 0%).

In PFS analysis, the original apparent heterogeneity (*P* = .06, *I*^2^ = 46%) disappeared after excluding the study by Brahmer^[16]^ (*P* = .32, *I*^2^ = 15%), which included only patients with squamous cell carcinoma. Thus, a lack of patients with non-squamous cell carcinoma led to heterogeneity in the subgroup analysis (Fig. 6). We believe that the classification of pathology was the main reason for the heterogeneity observed in PFS analysis.

### 3.7 Safety

**Figure 7:**
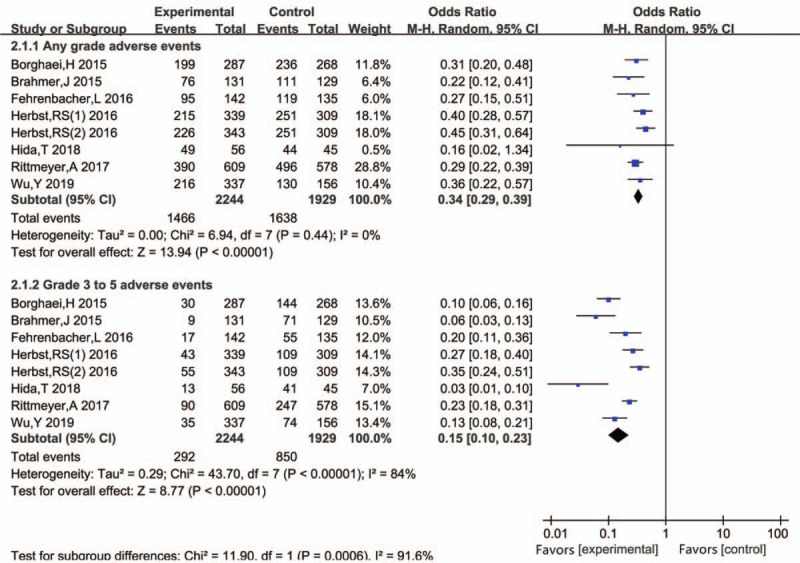
Hazard ratios of any grade adverse events (AEs)/grades 3–5 AEs for patients in the intervention and control groups. The effect of the therapy was calculated using a random-effects model.

A statistical analysis of 7 trials^[15–21]^ using a forest plot (Fig. 7) to describe the number of patients with side effects showed that the number of any grade side effects in the experimental group was significantly lower than that in the control group (HR 0.34, 95%CI 0.29–0.39, *P* < .001). Moreover, no heterogeneity was observed between the groups in this regard (*P* = .44; *I*^2^ = 0%).

The number of grade ≥3 side effects in the experimental group was also significantly lower than that in the control group (HR 0.15, 95%CI 0.10–0.23, *P* < .001). However, as assessed by random-effects model, there was still obvious heterogeneity (*P* < .001; *I*^2^ = 84%). This heterogeneity was possibly observed because 2 of the 7 trials^[17,18]^ reported grades 3 to 5 side effects, whereas the other 5 RCTs reported only the number of patients with grades 3 to 4 side effects.

### 3.8 Publication bias analysis

**Figure 8:**
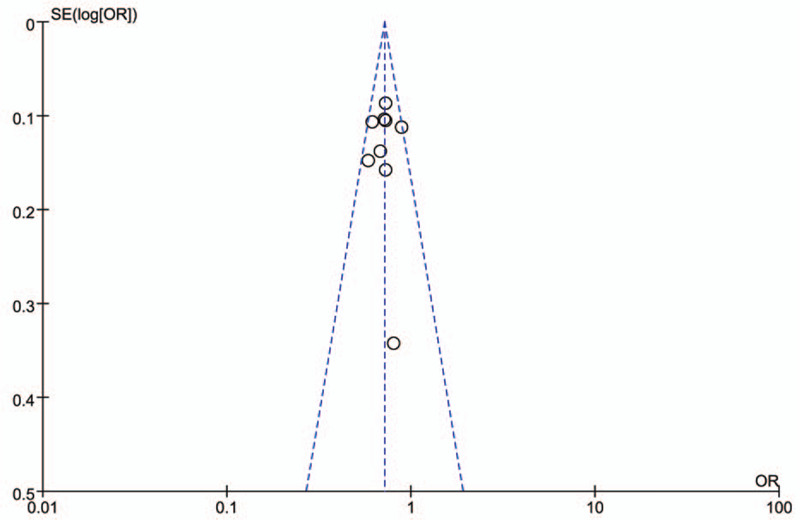
The Begg funnel plot of 8 included trials.

A visual inspection of the Begg funnel plot showed some asymmetry (Fig. 8). All 8 trials were at the left side of the line (OR = 1), indicating that there may be publication bias.

## 4 Discussion

At present, many clinical trials have shown that for treating patients with advanced NSCLC, pembrolizumab in combination with chemotherapy, as a first-line treatment, has a significantly better curative effect than chemotherapy alone.^[22–26]^ However, another study has shown that PD-L1 inhibitors, such as nivolumab, combined with chemotherapy, when used as the first-line treatment for NSCLC, have a high rate of discontinuation owing to treatment-related adverse events (approximately 21%). The adverse events resulting from the combined use of PD-L1 inhibitors and platinum-based doublet chemotherapy cannot be ignored.^[5]^ Notably, in recent years, platinum chemotherapy has been used as the first-line therapy for advanced NSCLC patients without target-driven gene mutations,^[27]^ but the problem of the high incidence of adverse reactions is difficult to address.^[28]^ PD-L1 inhibitors when used in combination chemotherapy result in serious side effects and high drug withdrawal rate. In addition, a report gas demonstrated that the incidence of grades 3 and 4 immune-related adverse events following immunosuppressant treatment is higher than that reported in clinical trials.^[6]^ Currently, it is critical to understand whether immunosuppressant drugs can prevent serious adverse events caused by chemotherapy when they are used alone as second- or later-line treatment and to ensure that they still have a good curative effect and are safe for patients.

This meta-analysis is to investigate and analyze the latest RCT evidence regarding the efficacy and safety of PD-L1 inhibitors alone with those of chemotherapy for treating advanced NSCLC. During the article screening process, some of the excluded literature was of high quality and highly consistent with screening criteria, but they allowed chemotherapy arm patients to receive immunotherapy in the event of disease progression.^[29]^ Although the complexity of clinical trials is taken into account, the researchers have to loosen test standards in some cases. However, in strict accordance with the statistical analysis, we believe that even if the patients meet the safety standards, the progress of the disease should be counted as the number of outcome indicators in the current treatment group, and should not receive immune checkpoint blockade treatment. This will lead to a large degree of deviation on the overall results. Through the analysis of the final 8 high-quality RCTs, enrolling more than 4000 patients with advanced NSCLC, we revealed that PD-L1 inhibitors, when used alone as second-line or later-line treatment, can effectively improve the OS and PFS compared with chemotherapy. Moreover, we noted that the ORR was also better following PD-L1 treatment than following chemotherapy. Furthermore, as determined using TPS, both PD-L1-positive and -negative patients benefit more from PD-L1 inhibitors than from traditional chemotherapy. Similarly, the efficacy of PD-L1 inhibitors in patients with squamous cell carcinoma and those with non-squamous cell carcinoma is also better than that of chemotherapy. However, our analyses failed to demonstrate if the tumor pathology and TPS score of tumors can be used as indicators of treatment selection for advanced NSCLC. Regarding safety, PD-L1 inhibitor treatment was associated with significantly fewer adverse effects than chemotherapy.

Irrespective, this study has some limitations. First, although all 8 trials were found to have a low risk of bias regarding blinding of participants, no studies used the blinding method during intervention. This is because of the inevitability of not being able to follow blinding when performing a subgroup analysis for assessing the effect of TPS and tumor pathology on the efficacy of PD-L1 inhibitors. In addition, randomly grouping patients according to TPS or tumor pathology will result in new intergroup differences, leading to a patients inconsistent baseline level of the study, which will affect the results. Second, most studies included in this meta-analysis enrolled European and American patients, and only 2 studies enrolled Asian patients,^[19,21]^ which may have led to a racial bias. Therefore, the results can only be generalized to European and American patients. Third, the assessment of publication bias was not convincing enough owing to the inclusion of only few articles. Therefore, further studies using more large-scale and high-quality RCTs are needed.

This meta-analysis aimed to conduct a comprehensive and strict search of clinical trials that met appropriate standards. It showed that PD-L1 inhibitors possess significant efficacy and safety as a second-line and later-line therapies for patients with advanced NSCLC. For patients with poor health status caused by advanced disease or first-line treatment-related side effects, PD-L1 inhibitors alone can effectively reduce the risk of mortality and improve the quality of life without the requirement for chemotherapy, which causes considerable toxicity and adverse effects. We believe that the results of this meta-analysis will help improve clinical treatment plans and provide new treatment options for patients with advanced NSCLC.

## Author contributions

WD and HS developed the study concept. All authors contributed to the study design. LW, DZ, and JM participated in literature searching and data extraction. LW, JM, and TW conducted the data analysis. LW and DZ interpreted the results and drafted the paper under the supervision of WD and HS. All authors approved the final version of the paper for submission.

**Conceptualization:** Lin-guang-jin Wu, Hua Sui, Wan-li Deng.

**Data curation:** Lin-guang-jin Wu, Dan-ni Zhou, Jun-zhi Ma.

**Formal analysis:** Dan-ni Zhou, Ting Wang.

**Investigation:** Jun-zhi Ma.

**Software:** Dan-ni Zhou.

**Validation:** Wan-li Deng.

**Visualization:** Hua Sui, Wan-li Deng.

**Writing – original draft:** Lin-guang-jin Wu.
